# Optimization and identification of bound polyphenols from rice bean (*Vigna umbellata*) skin dietary fiber, antioxidant activities and *α*-glucosidase inhibition mechanism

**DOI:** 10.1016/j.fochx.2025.103278

**Published:** 2025-11-08

**Authors:** Jing Liang, Jiayan Xie, Yue Gu, Jianhua Xie, Yi Chen, Bing Zheng, Yue Qiu, Xiaobo Hu, Qiang Yu

**Affiliations:** State Key Laboratory of Food Science and Resources, China-Canada Joint Laboratory of Food Science and Technology (Nanchang), Key Laboratory of Bioactive Polysaccharides of Jiangxi Province, Nanchang University, 235 Nanjing East Road, Nanchang 330047, China

**Keywords:** Rice bean, Dietary fiber, Bound polyphenols, *α*-Glucosidase, Inhibition mechanism, Molecular docking

## Abstract

*Vigna umbellata* is an important homologous medicinal and food legume, and its fruit is rich in polyphenols. Acidic, alkaline (highest yield) and enzymatic methods were used to release bound polyphenols from rice bean skin dietary fiber. 44 compounds were identified from the alkaline hydrolysis products. With the increase of polyphenol concentration within a specific range, the scavenging rate of DPPH• and ABTS^+^• radicals and the reduction ability of ferric ions were increased. Rice bean skin dietary fiber bound polyphenols (RBSDF-BP) showed significant *α*-glucosidase inhibitory activity. The results showed that Hydrogen bonding and hydrophobic forces were the binding forces for RBSDF-BP to form a stable polyphenol-enzyme complex with *α*-glucosidase. Molecular docking revealed that the three polyphenols of RBSDF-BP had a strong affinity for the active site in the hydrophobic cavity of the enzyme. This study provides valuable discoveries for the potential application of RBSDF-BP as natural inhibitors of *α*-glucosidase.

**Chemical compounds:**

Protocatechuic acid (PubChem CID: 72); Malic acid (PubChem CID: 525); Isoferulic acid (PubChem CID: 736186); p-Hydroxybenzoic acid (PubChem CID: 135); Citric acid (PubChem CID: 311), (+)-Catechin (PubChem CID: 9064), Fumaric acid (PubChem CID: 444972), Gallic acid (PubChem CID: 370), Azelaic acid (PubChem CID: 2266), (−)-Epicatechin (PubChem CID: 72276).

## Highlights

• Alkaline hydrolysis contained the highest total phenolic content and detected an abundance of phenolic acid compounds.
• The RBSDF-BP exhibited prominent antioxidant activity on DPPH•, ABTS^+^• and FRAP.
• The binding of RBSDF-BP shifted the structure of *α*-glucosidase to a more stable conformation.
• Epicatechin (the main flavonoid of RBSDF-BP) showed the highest affinity for *α*-glucosidase.

## 1 Introduction

Polyphenols are naturally produced by plants as secondary metabolites and are strong antioxidants that protect plants from oxidative stress. Several studies have shown that phenolic compounds can inhibit *α*-glucosidase activity and reduce the hydrolysis of carbohydrates, thus potentially controlling blood sugar (Aleixandre et al., 2022). Phenolic compounds usually exist in both free and bound forms. Although there is a tendency to report free phenolics in the literature, a large number of bound polyphenols remaining in solid dietary fibers after extraction with various combinations of aqueous and organic solvents have been neglected (Adom & Liu, 2002). Compared to extractable phenolics, some studies have shown that bound polyphenols have a higher potential for activity and health benefits (Luo et al., 2016). Additionally, insoluble dietary fiber (IDF) bound to polyphenols also benefits humans. IDF slows glucose absorption through its porous structure and produces short-chain fatty acids via fermentation by gut microbiota to regulate metabolism, thereby exerting a preventive effect against type II diabetes (Zhang et al., 2024). IDF not only regulates appetite and intestinal function by enhancing satiety and promoting bowel movements, but also exhibits beneficial physiological activities such as reducing oxidative stress and anti-inflammatory effects (Zheng et al., 2024).

Acidic, alkaline and enzymatic methods of hydrolysis have been used to release bound polyphenols. Among them, the alkali treatment method is more likely to effectively break the ether and ester bonds attached to polysaccharides, and acid hydrolysis mainly breaks the glycosidic bond, leaving the ester bond intact. Enzymatic decomposition occurs under mild conditions, avoiding degradation of phenolic acids due to excessive and low pH or high temperatures. Finding the optimal method for preparing bound polyphenols is of great significance for the development and utilisation of rice bean resources.

*α*-glucosidase (*α*-glu), located in the small bowel, acts on the hydrolysis of long-chain carbohydrates to produce monosaccharides, resulting in high blood glucose levels, and is considered to be one of the key enzymes in the treatment of type II diabetes. A number of *α*-glu inhibitors control elevated postprandial blood glucose levels by delaying intestinal absorption of glucose, notably acarbose, miglitol and voglibose. The use of these drugs is usually accompanied by adverse reactions such as diarrhea, abdominal pain and flatulence in the human body. Therefore, it is necessary to explore natural products with inhibitory enzyme effects and low side effects. Phenolic acids such as ferulic acid, caffeic acid and cinnamic acid, flavonoids such as kaempferol, myricetin and quercetin, and other polyphenol extracts have been shown to be potential *α*-glu inhibitors (Lim et al., 2022; Yu et al., 2024).

Rice bean (*Vigna umbellata* (Thunb.) Ohwi et Ohashi) is considered an underutilised legume crop of high value in terms of nutrient content and production potential. Bioactive substances such as various flavonoids and phenolic acids were detected in rice bean in addition to essential nutritional factors (Yao et al., 2012). Due to its tough skin, long cooking time and poor processing methods, rice beans skin are often discarded as processing by-products. However, most of the phenolic compounds are concentrated in the seed coat of legumes. Some studies have found that rice bean extracts have significant *α*-glu inhibitory activity, reflecting its potential to inhibit blood glucose in diabetic patients (Katoch et al., 2023). Although numerous studies have confirmed the activities of various bound polyphenols, in-depth exploration of the molecular mechanisms underlying their interactions with *α*-glu remains relatively scarce. The application of the inhibitory effect of polyphenols on *α*-glu needs to be preceded by exploring the mechanism of their interaction. To our knowledge, there are currently no specific reports on the mechanism of *α*-glu inhibition by RBSDF-BP.

In this study, we optimized the extraction process for bound polyphenols from rice bean skin dietary fiber. The extracted compounds were identified and quantified using UPLC-ESI-QTOF-MS/MS and UPLC-ESI-QqQ-MS/MS techniques. Subsequently, their antioxidant activities were evaluated in vitro through chemical methods. The inhibition mechanism of RBSDF-BP on *α*-glu was elucidated via inhibition kinetics analysis, fluorescence spectroscopy, circular dichroism, and molecular docking simulations. This study provides valuable insights into the development of potential natural active ingredients for hyperglycemia control and enhances the health value of rice bean skin.

## 2 Materials and methods

### 2.1 Materials and chemicals

The rice bean used as raw material for the experiment was obtained from Xuancheng, Anhui Province. The rice bean was peeled manually, crushed into powder with a crusher, and then sieved with 80-mesh sieve.

Protease, heat-stable *α*-amylase (2100 U/g), glucoamylase (100,000 U/mL), Folin-Ciocalteu reagent, DPPH• and ABTS^+^• were purchased from Aladdin Biotechnology. *α*-glucosidase (50 units/mg protein yeast), 4-nitrophenyl *α*-D-glucopyranoside (PNPG), and all standard reference materials including protocatechuic acid, phydroxybenzoic acid, malic acid, isoferulic acid, (+)-catechin, gallic acid, and epicatechin were products of Shanghai yuanye Bio-Technology Co., Ltd. All other chemicals were AR grade chemicals.

### 2.2 Preparation of rice bean skin dietary fiber (RBSDF)

Briefly, 100 g of rice bean skins were dissolved in 850 mL of water and then enzymatically digested by adding heat-resistant *α*-amylase (0.4 g), 0.2 % papain (0.5 g) and glucoamylase (3 mL), respectively. After adding the first enzyme, the reaction system was maintained in a 60 °C water bath with agitation for 0.5 h prior to the addition of the subsequent enzyme. After being centrifuging for 15 min at 4800*g* using a medium-speed centrifuge (TDL-5-A; Anting Scientific Instrument Factory, Shanghai, China), the substrate that had been washed with fivefold volume ultrapure water, 95 % and 70 % ethanol was vacuum filtered and lyophilized and placed in a freezer at −20 °C for storage.

### 2.3 Optimization of three methods to release bound polyphenols

Single-factor experimental design of alkaline, acidic, and enzymatic hydrolysis of bound polyphenol release in rice bean skin dietary fiber (RBSDF) was exhibited in Table S1, S2, S3. Briefly, RBSDF (5.0 g) was put in a 100 mL glass beaker and mixed with several NaOH, H_2_SO_4_, enzyme (pectinase and cellulase, mass ratio 1:2, pH 4) concentrations and several time/liquid-solid ratio on a 37 °C thermostatic shaker. The solution was neutralized to pH 2 with 8 mol/L NaOH/HCl solution, followed by the addition of twice the volume of ethanol and centrifugation at 4800*g* for 15 min, and finally the supernatant was collected for the determination of phenolic contents. Three independent replicates and three replicate analyses were performed.

Response surface methodology was used to determine the parameters affecting polyphenol extraction using Box-Behnken design approach. Concentration-X1, liquid-solid ratio-X2 and time-X3 were used as independent variables and total polyphenols content (Y) was the response variable.

### 2.4 Qualitative study of RBSDF-BP with UPLC-ESI-QTOF-MS/MS

A UHPLC30A system (Shimadzu, LC20AD, Kyoto, Japan), coupled with a Trip TOF™ 5600+ system (AB SCIEX, Foster City, California, USA) was used for analyzing the composition of RBSDF-BP. Separation was carried out in an Inertsil ODS-3 C18 column (2.1 mm × 100 mm, 1.8 μm). The mobile phase was typically composed of 0.1 % formylic acid in deionized water (A) and acetonitrile (B). The mobile phase flow rate was 0.30 mL/min, the injection volume was 5 μL, and the B phase varied as follows: 5–45 %, 0–15 min, 45–95 %, 15–17 min, 95–95 %, 17–18 min, 95–5 %, 18–18.1 min, 5–5 %, 18.1–22 min. The ESI source was operated in the negative ion pattern, and full scan mass spectral data was obtained from *m/z* 100 to 1000. UV spectrum was acquired in the 190–600 nm range and the diode array detector at 254, 280, 325 and 520 nm was used. The optimum values of the source parameters were as follows: capillary voltage, + 4.5 kV; drying gas flow, 11.0 L/min; drying gas temperature, 550 °C; atomizing gas pressure, 50 psi. The compounds in the bound polyphenols were discriminated by means of contrasting with the literature, relevant standards, or specialized databases.

### 2.5 Quantitative study of RBSDF-BP with UPLC-ESI-QqQ-MS/MS

The Agilent 1290II-6470 UPLC system (Agilent Technologies, America) equipped with a Zorbax Eclipse Plus C18 column (2.1 mm × 50 mm, 1.8 μm, Agilent Eclipse) was used for quantification. The mobile phase (phase A, 0.1 % formylic acid; phase B, acetonitrile) was selected at 0.30 mL/min flow velocity and 5 μL injection quantity, and phase A elution procedure: 95–90 % (0–2 min), 90–70 % (2–6 min), 70–55 % (6–9 min), 55–20 % (9–11 min), 20–0 % (11–13 min), 0–0 % (13–18 min), 0–95 % (18–22 min), 95–95 % (22–24 min). The optimum operating parameters were mainly as follows: capillary voltage of 4.5 kV, drying gas (N_2_) flow rate of 5 L/min, temperature of 350 °C; column oven temperature of 35 °C. The MassHunter optimiser was utilized in optimising the fragmenter voltage and collision energy for the target compounds. Quantification of each component was performed using Quant-my-way software to select different concentrations to construct the corresponding standard curves.

### 2.6 Antioxidant activity of RBSDF-BP

#### 2.6.1 DPPH• scavenging capacity assay

The antioxidant activity of RBSDF-BP was evaluated by DPPH method. 0.1 mmol/L DPPH radical solution was dissolved in ethanol, extracts were diluted to 2.5–100 μg/mL, 50 μL of RBSDF-BP/ascorbic acid and 150 μL of DPPH were mixed in 96-well plate. After incubation for 30 min at 25 °C avoiding light, the absorbance was detected at 517 nm using microplate reader (Thermo).

#### 2.6.2 ABTS^+^• scavenging capacity assay

In brief, the 7 mmol/L ABTS solution and the 2.45 mmol/L potassium persulfate stock solution were mixed (1:1) and was allowed to stand for approximately 14 h protected from light before dilution. The ABTS solution was diluted to an absorbance of 0.70 at 734 nm and 140 μL solution was extracted and mixed with RBSDF-BP (60 μL). The absorbance was measured at 734 nm after incubation for 5 min avoiding light.

#### 2.6.3 Ferric ion reducing antioxidant power (FRAP) assay

The FRAP reaction working solution was 10 mmol/L TPTZ solution with the addition of 300 mM CH₃COONa buffer solution (pH 3.6), 20 mM Fe^3+^ chloride solution and 40 mmol/L HCl in a volume ratio of 10:1:1. The RBSDF-BP (20 μL) was mixed with the FRAP reagent (180 μL). After incubation for 10 min at 37 °C, the absorbance was detected at 593 nm.

### 2.7 *α*-Glu inhibition study in vitro

#### 2.7.1 Inhibitory capacity of RBSDF-BP on *α*-glu

Method detailing the inhibitory activity of *α*-glu had been reported, with slight modifications (Sun et al., 2022). In brief, 260 μL of PBS (0.10 M), 20 μL of purified product (0, 8, 16, 32, 64, 128, 256, 512, 1024 μg/mL) with 0.1 % DMSO as solvent, and 20 μL of 2 U/mL *α*-glu were mixed (37 °C, 10 min). After that, 100 μL of 0.1 mM PNPG was introduced to react for 10 min, and the reaction system was terminated by adding 400 μL of 15 % volume fraction of Na_2_CO_3_. The absorbance of the reaction solution at 405 nm was read by a microplate reader (Thermo, Variosken Flash, USA).

(1) $$\mathrm{Inhibition rate}=\left(1-\frac{A_{3}-A_{2}}{A_{1}-A_{0}}\right)\times 100\%$$

A_0_, A_2_ represent the absorbance values of PBS and RBSDF-BP without enzyme; A_1_, A_3_ are the absorbance values of PBS and RBSDF-BP containing enzyme.

#### 2.7.2 Kinetic study of enzymatic inhibition

Reversible inhibition can be determined by plotting the enzyme concentration (U/mL) as the horizontal coordinate and the initial reaction rate (ΔOD/min) as the vertical coordinate, and determining whether the line passes through the origin. The initial reaction rates of *α*-glu with added polyphenols (0, 32, 64, and 128 μg/mL) were determined at different concentrations (0.5, 1, 1.5, 2, 2.5, 3 U/mL), respectively, and the concentration of the substrate PNPG (0.1 mmol/L) was fixed.

In contrast, the *α*-glu concentration (2 U/mL) was fixed and the type of inhibition was confirmed by obtaining the Lineweaver-Burk equation. Different concentrations of polyphenols (0, 32, 64 and 128 μg/mL) were reacted with six concentrations of substrates (0.1, 0.15, 0.2, 0.25, 0.3, 0.35 mmol/L PNPG) for 10 min at 37 °C, and the rate of the enzymatic reaction (1/V) was determined. The maximum initial reaction rate (V_max_) was obtained from the intercept of the Lineweaver-Burk plot, and the Michaelis constant (K_m_) was calculated from the slope of the equation.

(2) $$\frac{1}{V}=\frac{1}{V_{\max}}+\frac{K_{m}}{V_{\max}}\frac{1}{\left[I\right]}$$

where, [I] is the substrate concentration.

#### 2.7.3 Intrinsic fluorescence spectrometry

Specific amounts of *α*-glu (2.8 mL, 0.5 U/mL) were incubated with 1.4 mL of RBSDF-BP (0, 0.5, 1, 2, 3, 4, 5, 6, 7 μg/mL) for 0.5 h at temperatures (298.15 K, 304.15 K, 310.15 K). The emission wavelength ranged from 300 to 500 nm and the excitation was wavelength typically 280 nm, the slit width was 5 nm. The method used a fluorescence spectrometer (F-7000, Hitachi) and the corresponding blank solution (PBS) fluorescence values were subtracted to correct the fluorescence spectral data. The quantity of binding sites, fluorescence quenching parameter and binding constant were calculated using Stern-Volmer curve and double logarithmic curve. The equations are given below:

(3) $$\frac{F_{0}}{F}=1+K_{q}\tau_{0}\left[Q\right]=1+K_{\mathrm{sv}}\left[Q\right]$$

(4) $$\log\frac{F_{0}-F}{F}=\log K_{a}+\mathrm{nlog}\left[Q\right]$$

Where, [Q] and τ_0_ are the sample concentration and the lifetime of fluorophore, respectively; F and F_0_ represent the fluorescence intensities with or without RBSDF-BP; K_q_ and K_sv_ are bimolecular quenching rate constant and Stern–Volmer quenching constant, respectively; n and K_a_ are number of binding sites and the binding constant, respectively.

In order to deduct the inner filter effect of RBSDF-BP in fluorescence measurement due to its own UV absorption, the fluorescence intensity was corrected using the following formula. All fluorescence intensities used in this study are corrected values.

(5) $$F_{C}=F_{M}e^{\left(A_{1}+A_{2}\right)/2}$$

where F_C_ and F_M_ represent the calibration and measurement values of fluorescence intensity, respectively; A_1_ and A_2_ are the absorbance of RBSDF-BP at the excitation and emission wavelengths, respectively.

#### 2.7.4 Synchronous fluorescence spectrometry

The microenvironmental changes of tryptophan and tyrosine residues in *α*-glu were characterized using synchronous fluorescence spectroscopy. The wavelength intervals were set at Δλ = 15 nm (Tyr) and Δλ = 60 nm (Trp) and the excitation wavelength range was 200–500 nm. The ratio of synchronous fluorescence quenching (RSFQ) was calculated from the synchronous fluorescence spectra using the equation:

(6) $$\mathrm{RSFQ}=1-\left(\frac{F}{F_{0}}\right)$$

Where, F and F_0_ represent the synchronous fluorescence intensity of *α*-glu with and without RBSDF-BP.

#### 2.7.5 Surface hydrophobicity

Change in the surface hydrophobicity of RBSDF-BP with *α*-glu was observed using a fluorescent probe approach according to the method described by previous authors but with minor modifications (Dai et al., 2018). Briefly, the reaction system consisted of 2.8 mL *α*-glu (0.5 U/mL), 1.4 mL RBSDF-BP (0–7 μg/mL), and 20 μL ANS (1 mM). The emission fluorescence spectra of the mixture from 400 to 700 nm were then recorded employing a fluorescence spectrometer at an excitation wavelength of 370 nm and excitation and emission slit widths of 5 nm. Measurements were made and appropriate blanks were subtracted to rectify for fluorescence background.

#### 2.7.6 Circular dichroism (CD)

Circular dichroism spectrum was recorded using CD spectrometer (Bio-Logi, MOS-450, France). Before performing the measurements, 1.5 mL of RBSDF-BP solution with different concentration gradients (40, 80, 120 μg/mL) was taken and mixed with an equal amount of 8 U/mL of *α*-glu, and then incubated for 30 min. The background signals of the corresponding RBSDF-BP and PBS solutions were subtracted. The spectra of the mixture were recorded in a 1-mm optical path under nitrogen protection, and 850 μL of the solution to be measured was taken each time for detection. The scanning wavelength was 190–250 nm and the slit width was 2 nm. The results were professionally analysed on dichroweb.

#### 2.7.7 Molecular docking

Molecular docking techniques were used to characterize the interaction pattern between RBSDF-BP and *α*-glu. In this research, the top 10 polyphenols in the content of RBSDF-BP were selected as ligands as polyphenol samples and the magnitude of binding energy between them was compared. The sequence identity of *Saccharomyces cerevisiae α* -glucosidase (GI No: 411229) and isomaltase (PDB ID: 3A4A) was 72.51 % (Xu et al., 2024). So we downloaded the 3D structure of *α*-glu (PDB ID: 3A4A) on the Protein Data Bank and stripped the water molecule and the original ligand. Then, the 3D configurations of 10 polyphenol-ligands were obtained from the PubChem Database and the files were made to be converted into PDB format in Chem3D 21.0.0 software. Before molecular docking, the structures of proteins and ligands were hydrogenated, torsion bonds and centers were detected, charges were calculated, and charges were distributed using AutodockTools 1.5.7 software. After that, after 20 dockings, the result with the lowest binding energy was chosen as the final analysis. Finally, the best docked conformations were inputted into PyMOL for visualization.

### 2.8 Statistical analysis

Three parallel experiments were conducted for all experiments, and the results were expressed as mean ± standard deviation. Statistical analyses were performed using IBM SPSS statistical 27 software (SPSS Inc., Chicago, IL, USA). To determine the statistical significance of differences between groups, the parametric dataset was tested using Duncan's multiple range test. In addition, *P* < 0.05 was considered statistically significant.

## 3 Results and discussion

### 3.1 Optimization for the release of RBSDF-BP in three methods

**Fig. 1:**
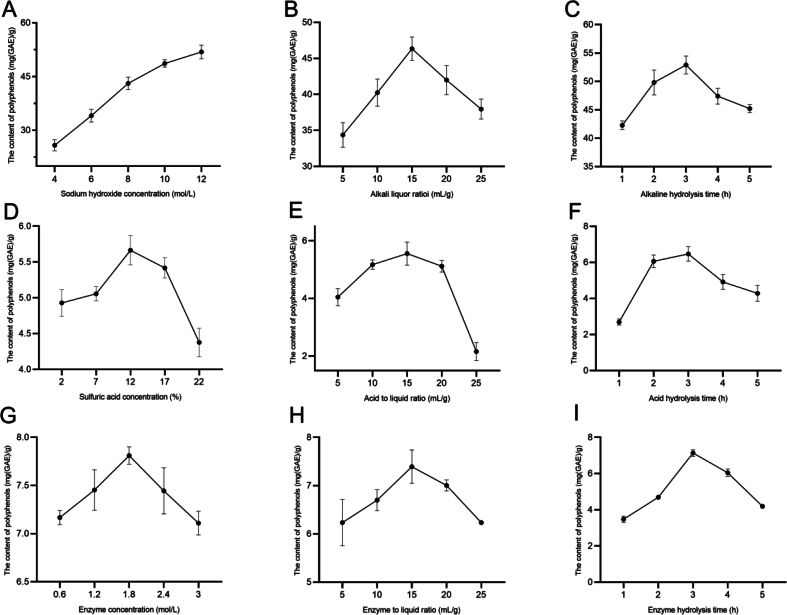
Impact of several factors on the dietary fiber bound polyphenol total phenolic content of rice bean skins. (A) Impact of sodium hydroxide concentration on TPC. (B) Impact of alkaline liquor ratio on TPC. (C) Impact of alkaline hydrolysis time on TPC. (D) Impact of H_2_SO_4_ concentration on TPC. (E) Impact of acid-liquid ratio on TPC. (F) Impact of acid hydrolysis time on TPC. (F) Impact of enzyme concentration on TPC. (G) Impact of enzyme-liquid ratio on TPC. (I) Impact of enzymatic time on TPC.

Based on the results of the single factor of the three methods (Fig. 1), the optimal conditions for the Box-Behnken design with alkaline hydrolysis as the center point of the experiment are shown in Table 1, and the optimal conditions for acidic and enzymatic hydrolysis are shown in Table S4–5, and Box-Behnken design flowchart is shown in Fig.S3. The equations of polyphenol extraction in relation to the concentration of NaOH (X_1_), the liquid-to-solid ratio (X_2_), and the extraction time (X_3_) were obtained from the regression model as follows:

$$Y=50.05+3.65X_{1}+1.64X_{2}–1.70X_{3}+1.83\;X_{1}X_{2}+1.75\;X_{1}X_{3}+0.1382X_{2}X_{3}–1.63{X_{1}}^{2}-2.28{X_{2}}^{2}-2.99{X_{3}}^{2}$$

**Table 1:** Design of Box-Behnken experiments in alkaline hydrolysis.

Number	NaOH concetration (X1)	Liquid-solid ratio (X2)	time (X3)	TPC (Y)
1	8(−1)	10(−1)	3(0)	43.173
2	12(1)	10(−1)	3(0)	45.887
3	8(−1)	20(1)	3(0)	42.735
4	12(1)	20(1)	3(0)	52.781
5	8(−1)	15(0)	2(−1)	44.870
6	12(1)	15(0)	2(−1)	49.614
7	8(−1)	15(0)	4(1)	37.758
8	12(1)	15(0)	4(1)	49.489
9	10(0)	10(−1)	2(−1)	44.855
10	10(0)	20(1)	2(−1)	47.899
11	10(0)	10(−1)	4(1)	41.396
12	10(0)	20(1)	4(1)	44.993
13	10(0)	15(0)	3(0)	49.141
14	10(0)	15(0)	3(0)	49.520
15	10(0)	15(0)	3(0)	50.829
16	10(0)	15(0)	3(0)	51.011
17	10(0)	15(0)	3(0)	49.773

Where Y was the total polyphenol content (TPC) (mg GAE/g IDF), X_1_, X_2_ and X_3_ were the NaOH volume fraction (%), liquid-solid ratio (mL/g) and extraction time (h), respectively.

**Fig. 2:**
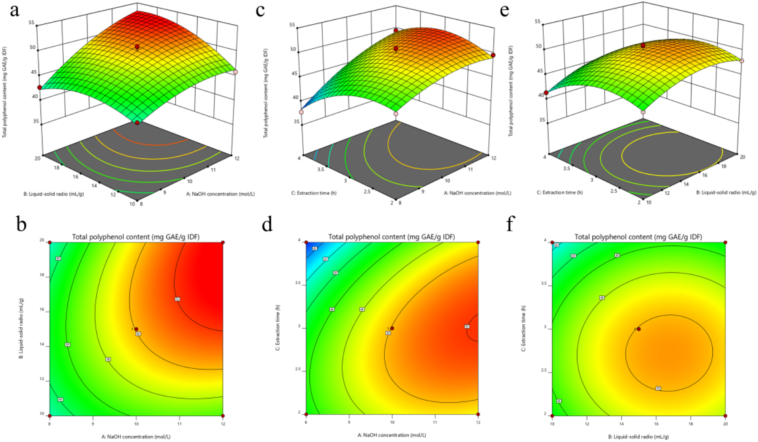
Response surface plots. NaOH concentration and liquid-solid ratio (a and b). NaOH concentration and extraction time (c and d). Liquid-solid ratio and extraction time (e and f).

The regression models were both significant and had high F-values (> 149.32) as shown in Table 2 and Tables S6–7. The R^2^ of the responses were greater than 0.9825 and the R^2^_adj_ were all close to R^2^ and nearly 1, indicating an accurate model fit. The coefficient of variation (< 10 %) substantiated the model's dependability and repeatability. The validity of the model was verified using a non-significant *p*-value for lack of fit (*p* > 0.05), indicating that the model accurately predicted change. The steep slopes indicate significant interactions between the parameters (Fig. 2 and Fig. S1–2). Since the target point (maximum) was located in the center of the area, the contour map showed a circular or elliptical state (Fig. S1). The surface plots in Fig. S1 d-f were steeper, with a significant interaction between X1-X3 and X2-X3. As depicted in Fig. 2 (d), the 2D surface plot was steeper, indicating a notable interaction between alkali concentration (X1) and hydrolysis time (X3).

**Table 2:** ANOVA for the quadratic response surface model in alkaline hydrolysis.

Source	Sum of Squares	df	Mean Square	F	p	
Model	255.52	9	28.39	43.79	< 0.0001	significant
X1	106.84	1	106.84	164.77	< 0.0001	
X2	21.44	1	21.44	33.07	0.0007	
X3	23.13	1	23.13	35.67	0.0006	
X1X2	13.44	1	13.44	20.73	0.0026	
X1X3	12.2	1	12.2	18.82	0.0034	
X2X3	0.0765	1	0.0765	0.1179	0.7414	
X1^2^	11.21	1	11.21	17.29	0.0042	
X2^2^	21.87	1	21.87	33.73	0.0007	
X3^2^	37.65	1	37.65	58.06	0.0001	
Residual	4.54	7	0.6484			
Lack of Fit	1.82	3	0.6082	0.8963	0.5165	not significant
Pure Error	2.71	4	0.6785			
Cor Total	16.71	16

The optimum value of content was acquired by regression equation solution. As depicted in Table 3 (53.13, 52.54 mg GAE/g IDF) and Tables S8–9, the predicted values were consistent with the actual results, indicating that the regression model was plausible. In summary, the quadratic regression model can be used to optimize and predict the release process of bound polyphenols. Following optimization, the extraction rates of total polyphenols by alkaline, acidic, and enzymatic hydrolysis increased to 2.6, 2.2 and 2.0 times their pre-optimization levels, respectively. This demonstrates that the optimized parameters significantly promoted polyphenol release, with the alkaline hydrolysis exhibiting the most pronounced enhancement. Under acid hydrolysis and enzymatic hydrolysis conditions, the peak total polyphenol yields reached 6.40 and 7.06 mg GAE/g IDF, respectively, both significantly lower than the extraction efficiency achieved by the alkaline hydrolysis. Since alkaline hydrolysis yielded the highest total polyphenol content in the crude extract, phenolic compounds were released better under alkaline conditions than under acidic conditions, reducing losses. Alkaline treatment was commonly used to extract bound phenolic acids from grains, so we chose the optimal alkaline extraction method as the standard for all the following experiments.

**Table 3:** The predicted and actual value of response in alkaline hydrolysis.

	NaOH concentration (mol/L)	Liquid-solid ratio (mL/g)	Extraction time (h)	Total polyphenol content (mg GAE/g IDF)
Optimum conditions (predicted)	11.84	17.44	3.19	53.13
Modified conditions (actual)	12.00	15.00	3.00	52.54

### 3.2 Qualitative study of RBSDF-BP with UPLC-ESI-QTOF-MS/MS

**Fig. 3:**
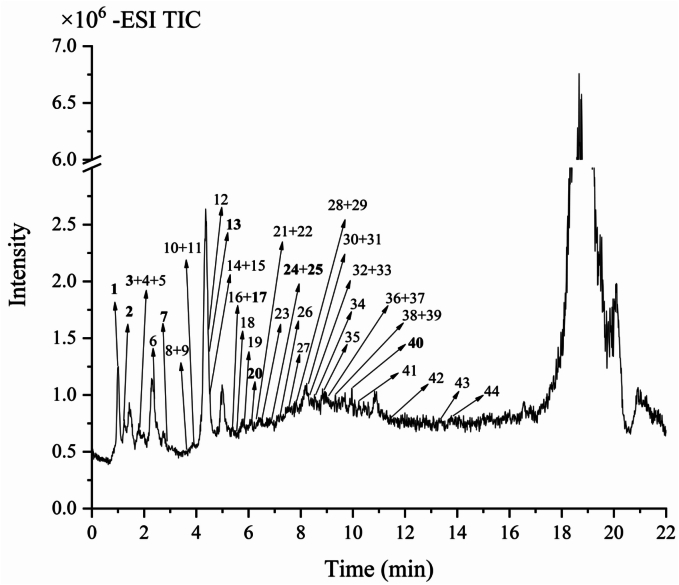
Total ion chromatogram of RBSDF-BP by alkaline hydrolysis under negative ion mode.

The crude extract obtained by alkaline hydrolysis was purified using ethyl acetate extraction method, and the pure RBSDF-BP was acquired as (42.42 ± 0.20)%. The identification of phenolic compounds was carried out by UPLC-ESI-QTOF-MS/MS by comparing the characteristic ion fragments (*m/z*), retention times (t_R_) and molecular formulae with online databases, published literature and standards. Table 4 listed the 44 major compounds initially identified. The total ion chromatograms of RBSDF-BP compounds extracted by alkaline hydrolysis on negative ionization pattern were displayed in Fig. 3.

**Table 4:** Characterization of RBSDF-BP.

Peaks	t_R_(min)	Formula	[M − H]^−^ (*m/z*)		Main fragmention (*m/z*)	Identification
			Measured	Predicted		
Phenolic acids and derivatives						
4	1.984	C_7_H_6_O_3_	137.0250	137.0244	119.0126	Hydroxybenzoic acid a
5	1.986	C_8_H_8_O_5_	183.0308	183.0371	139.0400,137.0255	DL-3,4-Dihydroxymandelic acid b
7	2.724	C_7_H_6_O_5_	169.0143	169.0145	125.0241,97.0277,79.0189	Gallic acid a, b, c
8	3.702	C_13_H_16_O_10_	331.0688	332.2610	169.0145,125.0257	Gallic acid hexoside a, b
9	3.764	C_8_H_8_O_4_	167.0349	167.0350	123.0433,121.0300	3-Hydroxymandelic acid a, b
10	3.927	C_9_H_10_O_5_	197.0451	197.0458	179.0342,135.0450,123.0461	Danshensu a
11	3.940	C_14_H_18_O_9_	329.0879	329.0878	167.0349	Woodorien a
12	4.222	C_8_H_6_O_5_	181.0145	181.0142	153.0195,135.0093	2-Hydroxyterephthalic acid a
13	4.247	C_7_H_6_O_4_	153.0194	153.0183	109.0292	Protocatechuic acid a, b, c
16	5.531	C_7_H_6_O_3_	137.0249	137.0251	109.0327	Protocatechuic aldehyde a
17	5.586	C_7_H_6_O_3_	137.0252	137.0244	93.9341	p-Hydroxybenzoic acid a, c
18	5.788	C_7_H_6_O_3_	137.0244	137.0244	93.0351,65.0388	Salicylic acid a, c
19	5.906	C_7_H_6_O_4_	153.0195	153.0193	109.0339	Dihydroxybenzoic acid a
20	6.185	C_10_H_10_O_4_	193.0511	193.0579	178.0327，134.0356	Isoferulic acid ^bc^
21	6.302	C_10_H_10_O_5_	209.0463	209.0455	165.0552	Hydroxyferulic acid a, b
23	6.517	C_8_H_8_O_4_	167.0349	167.0357	152.0154,108.0212	Vanillic acid a, c
26	7.36	C_16_H_18_O_9_	353.1174	353.1101	191.0402,173.0526,161.0256	Chlorogenic acid a, c
29	7.639	C_9_H_10_O_3_	165.0551	165.0629	137.0244	Ethylparaben b
30	7.941	C_11_H_12_O_5_	223.0606	223.0612	179.0297	Sinapic acid a, c
31	7.963	C_7_H_6_O_4_	153.0189	153.0193	107.0158,65.0023	Phloroglucinocarboxaldehyde a, b
32	8.003	C_9_H_10_O_5_	197.0464	197.0455	169.0140,124.0206	Ethyl gallate a, b, c
33	8.028	C_9_H_8_O_3_	163.0402	163.0401	119.0486,93.0347	p-Coumaric acid a
35	8.414	C_9_H_8_O_4_	179.0350	179.0350	135.0498	Caffeic acid a, b, c
37	8.632	C_10_H_10_O_4_	193.0502	193.0505	178.0278,149.0589,134.0380	Ferulic Acid a, c
38	8.786	C_9_H_8_O_5_	195.0301	195.0299	167.0418,136.0181,108.0224	4-Methoxyphthalic acid a
Flavonoids and derivatives						
24	7.022	C_15_H_14_O_6_	289.0373	289.0718	178.9927,151.0050	Epicatechin a, c
25	7.100	C_15_H_14_O_6_	289.0710	289.0718	245.0385,179.0023,109.0300	Catechin a, b, c
27	7.478	C_15_H_10_O_8_	317.0299	317.0264	191.0017,163.0038,125.0188	Myricetin a, b
34	8.311	C_27_H_30_O_16_	609.1464	609.1435	3,000,274	Rutin a, c
36	8.619	C_21_H_20_O_12_	463.0880	463.0875	301.0338,300.0271,271.0250	Quercetin-3-O-galactoside a, b, c
39	8.888	C_15_H_12_O_7_	303.0502	303.0510	285.0428,259.0603,241.0541	Taxifolin a, b, c
41	10.166	C_15_H_12_O_6_	287.0565	287.0561	259.0612,243.0613	Aromadendrin a, b
42	11.981	C_15_H_10_O_7_	301.0355	301.0361	151.0053,121.0300	Quercetin a, c
43	13.361	C_15_H_12_O_5_	271.0619	271.0684	151.0031,119.0497	Naringenin a, b, c
44	13.688	C_15_H_10_O_6_	285.0412	285.0405	239.0357,229.0491,211.0378	Kaempferol a, b, c
Organic acids						
1	1.254	C_4_H_6_O_5_	133.0141	133.0132	115.0045,71.0129	Malic acid a, c
2	1.29	C_4_H_4_O_4_	115.0030	115.0041	71.0491	Fumaric acid a, c
3	1.961	C_6_H_8_O_7_	191.0197	191.0197	162.8961,146.8994,111.0090,87.0110	Citric acid a, c
6	2.114	C_4_H_6_O_4_	117.0190	117.0193	73.0298	Succinic acid a
14	4.559	C_7_H_10_O_5_	173.0443	173.0455	111.0803,85.0646	Shikimic acid a
15	4.573	C_6_H_8_O_7_	191.0220	191.0197	111.0045,129.0363	2-Oxogluconic acid a
40	9.929	C_9_H_16_O_4_	187.0981	187.0976	125.0973,97.0658,57.0330	Azelaic acid a, c
Other compounds						
22	6.362	C_8_H_8_O_3_	151.0404	151.0401	136.0169,109.0299,108.0220	Vanillin a, c
28	7.606	C_9_H_6_O_4_	177.0191	177.0192	133.026	Esculetin a, b

a Comparison with literature.

b Comparison with mass bank.

c Comparison with standard.

#### 3.2.1 Phenolic acid and derivatives

Overall, 25 phenolic acid and derivatives were recognized in RBSDF-BP. Compounds 7 (*m/z* 169.0143), 23 (*m/z* 167.0349), 33 (*m/z* 163.0402) and 13 (*m/z* 153.0194) were identified as gallic acid, vanillic acid, *p*-coumaric acid and protocatechuic acid, respectively. Compound 19 (*m/z* 153.0195) showed fragment ion (frag-ion) with *m/z* 109.0339 [M-H-CO_2_]^−^, was characterized as dihydroxybenzoic acid; Chemical 31 (*m/z* 153.0193) produced frag-ion with *m/z* 107.0158 [M−H−CO−H_2_O]^−^, was identified as phloroglucinocarlboxaldehyde, both compounds are isomers of compound 14. Compound 12 (*m/z* 181.0506) displayed frag-ions with *m/z* 163.0043 [M−H−H_2_O]^−^, 153.0195 [M−H−CO]^−^ and 135.0093 [M−H−CO−H_2_O]^−^, was identified as 2-hydroxyphenyllactic acid (Xie et al., 2021). Compound 16 (*m/z* 137.0249), compound 18 (*m/z* 137.0252), compound 17 (*m/z* 137.0244) and compound 4 (*m/z* 137.0250) were all isomers and showed major frag-ions at characteristic ion fragments 109.0327 [M−H−H_2_O]^−^, 93.0351 [M−H−CO_2_]^−^, 93.0341 [M−H−CO_2_]^−^ and 119.0126 [M−H−H_2_O]^−^, respectively, were indicated as protocatechuic aldehyde, salicylic acid, *p*-hydroxybenzoic acid (PHBA) and hydroxybenzoic acid. Compound 38 (*m/z* 195.0301) displayed frag-ions with *m/z* 167.0418 [M−H−CO]^−^, 136.0181 [M−H−C_2_H_3_O_2_]^−^ and 108.0224 [M−H−C_3_H_3_O_3_]^−^, was considered as 4-methoxyphthalic acid. Chemical 21 (*m/z* 209.0463) was recognized as hydroxyferulic acid, showed frag-ion at *m/z* 165.0552 [M−H−CO_2_]^−^. Chemical 9 (*m/z* 167.0349) was the isomer of compound 23, presented frag-ions with *m/z* 121.0300 [M−H−HCOOH]^−^ and 123.0433 [M−H−CO_2_]^−^, was found to be 3-hydroxymandelic acid. The initial ions of chemicals 35 (*m/z* 179.0350) and 30 (*m/z* 223.0606) created frag-ions at *m/z* 135.0498 and 179.0297 through losing [M−H−CO_2_]^−^, were considered as caffeic acid and sinapic acid, respectively. Chemical 37 (*m/z* 193.0502), was considered as ferulic acid (isomer of compound 20) depended on the typical frag-ions *m/z* 178.0278 [M−H−CH_3_]^−^, 149.0589 [M−H−CO_2_]^−^, 134.0380 [M−H−C_2_H_3_O_2_]^−^. Compound 20 (*m/z* 193.0511) displayed frag-ions *m/z* 178.0327 [M−H−CH_3_]^−^ and 134.0356 [M−H−C_2_H_3_O_2_]^−^, was tentatively considered as isoferulic acid. Compound 5 (*m/z* 183.0308) displayed frag-ions at *m/z* 139.0400 [M−H−CO_2_]^−^, 137.0255 [M−H−CO−H_2_O]^−^, was tentatively identified as dl-3,4-dihydroxymandelic acid. Compound 10 (*m/z* 197.0451) and compound 32 (*m/z* 197.0464) exhibited isomerism and displayed major frag-ions with *m/z* 179.0342 [M−H−H_2_O]^−^, 135.0450 [M−H−CO_2_−H_2_O]^−^ and 169.0140 [M−H−H_2_O]^−^, respectively, were suggested as danshensu and ethyl gallate. Chemical 11 (*m/z* 329.0879) was recognized as woodorien by individually revealed [M−H−C_6_H_10_O_5_]^−^, generating notable fragment ions with *m/z* 167.0349. Compound 8 (*m/z* 332.261) showed frag-ions at *m/z* 169.0145 and 125.0257, was tentatively uggested as gallic acid hexoside. Compound 26 (*m/z* 353.1174) displayed frag-ions *m/z* 191.0402 [M−H−C_9_H_6_O_3_]^−^, 173.0526 [M−H−C_9_H_6_O_3_-H_2_O]^−^ and 161.0256 [M−H−C_7_H_10_O_5_−H_2_O]^−^, was recognized as Chlorogenic acid. Compound 29 (*m/z* 165.0551) showed frag-ions with *m/z* 137.0244 [M−H−CO]^−^, was tentatively recognized as Ethylparaben. Several distinctive phenolic compounds had been consistently detected in numerous natural byproducts. Caffeic acid, gallic acid, *p*-coumaric acid were identified in fermented *rosa roxburghii* pomace (Wang et al., 2024). Ferulic, vanillic, sinapic and chlorogenic acids were extracted from sugarcane tips (Huang et al., 2025).

#### 3.2.2 Flavonoid compounds and related derivatives

Ten flavonoids compounds and related derivatives were found in RBSDF-BP. Compounds 27 (*m/z* 317.0299), 25 (*m/z* 289.0710), 39 (*m/z* 303.0502), and 43 (*m/z* 271.0619) were characterized as myricetin, catechin, taxifolin and naringenin based on available literature (Huang et al., 2022). Compound 24 contained one precursor ion [M−H]^−^ with *m/z* 289.0373 and frag-ions with *m/z* 178.9927 [M−H−C_6_H_6_O_3_]^−^ and 151.0050 [M−H−C_7_H_8_O_3_]^−^, was characterized as epicatechin. Compound 42 (*m/z* 301.0355) was deprived of a unit [M−H−C_8_H_6_O_3_]^−^ in MS^2^ data for one frag-ion with *m/z* 151.0053 and was further deprived of a unit [M−H−H_2_O]^−^ for one frag-ion *m/z* with 121.0300, was characterized as quercetin. According to Mass Bank database and the accurate fragmentation patterns, compounds 36 (*m/z* 463.0880), compounds 41 (*m/z* 287.0565) and 44 (*m/z* 285.0412) were tentatively characterized to be quercetin-3-o-galactoside, aromadendrin and kaempferol, of which, as far as we know, there was a lack of previous literature on the former two substances with respect to rice bean components. Compound 34 (*m/z* 609.1464) with frag-ions at *m/z* 300.0274, was characterized as rutin, which was also found in ruc leaves, tobacco leaves, and orange peel (Amin et al., 2015).

#### 3.2.3 Organic acid compounds

A sum of 7 organic acids were found in RBSDF-BP. The characteristic frag-ions such as [M−H−H_2_O]^−^, [M−H−CO_2_]^−^, [M−H−CO_2_−H_2_O]^−^, were mainly generated. Compound 40 contained one precursor ion with *m/z* 187.0981 with a MS^2^ data at *m/z* 125.0973 [M−H−CO_2_−H_2_O]^−^ and 97.0658 [M−H−CO−CO_2_−H_2_O]^−^ was recognized as azelaic acid, and discovered in carrot bound polyphenols (Dong et al., 2021). Compounds 2 (*m/z* 115.003), 6 (*m/z* 117.0190), and 3 (*m/z* 191.0197) revealed [M−H−CO_2_]^−^ main frag-ions with *m/z* 71.0491, 73.0298, and 146.8994, were identified as fumaric acid, succinic acid, and citric acid, respectively. And compound 15 (*m/z* 191.0220) was the isomer of compound 3, indicated frag-ions with *m/z* 129.0363 [M−H−CO_2_−H_2_O]^−^ and 111.0045 [M−H−C_2_H_6_O_2_−H_2_O]^−^, was recognized as 2-oxogluconic acid. Compound 1 (*m/z* 133.0140) displayed the main fragment ion with *m/z* 115.0045 [M−H−H_2_O]^−^ and 71.0129 [M−H−CO_2_−H_2_O]^−^, which was recognized as malic acid refering to the reported literature (Xie et al., 2021). Compound 14 (*m/z* 173.0443) possessed a fragment ion (111.0803) with miss CO_2_ + H_2_O molecule, was recognized as shikimic acid.

#### 3.2.4 Other compounds

Compound 22 (*m/z* 151.0404) displayed characteristic frag-ions with *m/z* 136.0169 [M−H−CH_3_]^−^ and 108.0220 [M−H−CO_2_]^−^ was recognized as vanillin. Compound 28 (*m/z* 177.0191) displayed a characteristic frag-ion with *m/z* 133.026 [M−H−CO_2_]^−^ was recognized as esculetin.

### 3.3 Quantitative study of RBSDF-BP

The content of RBSDF-BP was measured with UPLC-ESI-QqQ-MS/MS (Table 5). In total, there were 17 major components containing 7 phenolic acids, 4 organic acids and 6 flavonoids. RBSDF-BP had a high phenolic acid content. The main phenolic acid was protocatechuic acid consisting of 799.50 μg/g DW, subsequently isoferulic acid (62.81 μg/g DW) and PHBA (29.22 μg/g DW). In addition, the other four major phenolic acids were gallic acid, ferulic acid, chlorogenic acid and vanillic acid, which ranged from 1.95 to 17.50 μg/g DW.

**Table 5:** Quantitative results of RBSDF-BP.

Compound	Regression equations	R^2^	Precursor ion	Product ion	Fragmentor voltage	Collision energy	Concentration
	(ng/mL)		(*m/z*)	(*m/z*)	(V)	(eV)	(μg/g DW)
Protocatechuic acid	y = 10.950× + 35,489	0.9966	153.1	109	102	16	799.50 ± 42.31
Malic acid	y = 9.3142×-133.31	0.9953	133.1	115	70	8	69.64 ± 4.05
Isoferulic acid	y = 0.9334× + 122.40	0.9973	193.2	134	87	16	62.81 ± 4.00
p-Hydroxybenzoic acid	y = 17.305× + 7445.8	0.9900	137.1	93.1	82	16	29.22 ± 1.27
Citric acid	y = 7.6061×-3768.2	0.9971	191.1	111	77	12	29.00 ± 3.26
(+)-Catechin	y = 2.7720× + 441.44	0.9938	289.3	245.1	151	16	25.31 ± 2.91
Fumaric acid	y = 2.6781×-672.11	0.9951	115.1	71.2	87	4	20.67 ± 0.78
Gallic acid	y = 24.594× + 1964.5	0.9977	169.1	125	114	12	17.50 ± 0.95
Azelaic acid	y = 21.452× + 6354.3	0.9950	187.2	125.1	102	12	10.19 ± 1.38
(−)-Epicatechin	y = 6.8870×-495.83	0.9991	289.3	245.1	151	16	9.83 ± 0.68
Ferulic Acid	y = 8.5200× + 472.94	0.9991	193.2	134	92	12	6.05 ± 0.08
Chlorogenic acid	y = 0.9019×-26.213	0.9965	353.3	191	188	20	4.17 ± 2.4
Quercetin-3-O-galactoside	y = 36.282×-567.80	0.9984	463.4	300.1	200	28	2.57 ± 0.08
Vanillic acid	y = 1.1331× + 22.330	0.9991	167.1	152	77	12	1.95 ± 0.15
Dihydroquercetin	y = 40.607× + 488.12	0.9996	303.2	285.1	156	8	1.94 ± 0.15
Rutin	y = 65.447×-847.04	0.9990	609.5	609.1	240	40	1.55 ± 0.04
Kaempferol	y = 1.0197× + 27.599	0.9962	285.2	195	161	28	1.10 ± 0.13

DW, dry weight of dietary fiber.

In passion fruit, protocatechin acid was also the most abundant polyphenols, followed by vanillic acid and ferulic acid (Shanmugam et al., 2018). This study also reported its potent *α*-glu inhibitory activity and antioxidant activity. In addition, gallic acid and protocatechuic acid were common phenolic acids found in legumes, both of which showed high levels in our study (Wang et al., 2016). It has been reported that isoferulic acid is one of the main phenolic acids in cereal alkaline extracts, which matches our results (Guo & Beta, 2013).

Flavonoids are mainly present in the bean seed coat in a bound form and are thought to contain mainly catechin, quercetin and rutin. Of the flavonoids found in the extracts, catechin (25.31 μg/g DW) and epicatechin (9.83 μg/g DW) were higher, subsequently came quercetin-3-O-galactoside (2.57 μg/g DW), dihydroquercetin (1.94 μg/g DW), rutin (1.55 μg/g DW) and kaempferol (1.10 μg/g DW). The four organic acids were malic, citric, fumaric and azelaic acid with concentrations of 69.64, 29.00, 20.67 and 10.19 μg/g DW, respectively.

### 3.4 Antioxidant activity of RBSDF-BP

**Fig. 4:**
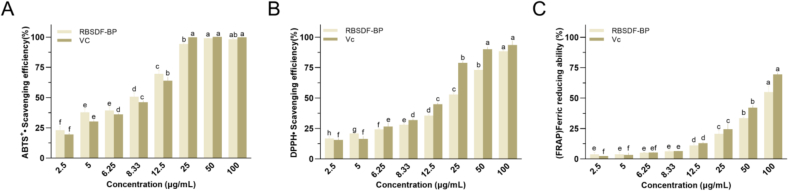
Antioxidant activities of RBSDF-BP. (A) ABTS^+^• radical scavenging activity. (B) DPPH• radical scavenging activity. (C) Ferric-reducing antioxidant power.

According to a large number of studies, the consumption of phenolic compounds present in the seeds of grain legumes acted as valuable antioxidants (Singh et al., 2017). As depicted in Fig. 4, the clearance rate gradually increased at concentrations ranging from 2.5 to 100 μg/mL.The ABTS radical cation (ABTS^+^•) assay could be used to assess the antioxidant activity of both fat soluble and water soluble antioxidants. The IC_50_ (Table 6) of RBSDF-BP and Vc was 7.281 and 8.211 μg/mL, respectively. Polyphenols and Vc showed similar IC_50_ values, indicating that RBSDF-BP had highly efficient ABTS^+^• free radical scavenging activity. At a concentration of 200 μg/mL, black bean polyphenol extract exhibited nearly 100 % ABTS^+^• free radical scavenging activity (Yang et al., 2024). In contrast, RBSDF-BP achieved comparable scavenging capacity at a significantly lower concentration (25 μg/mL), indicating that RBSDF-BP possesses substantially higher antioxidant activity than black bean polyphenol extract.

**Table 6:** Antioxidant activities of RBSDF-BP.

Item	IC_50_ value (μg/mL)		
	DPPH•	ABTS^+^•	FRAP
Vc standard	12.81 ± 0.56	8.21 ± 0.33	58.20 ± 1.47
RBSDF-BP	19.80 ± 1.04	7.28 ± 0.40	86.39 ± 1.85

Since the DPPH radical is a colored and relatively stable nitrogen center radical, it was frequently used to analyze the antioxidant capacity of phenols and other natural compounds. As shown in Table 6, The IC_50_ of RBSDF-BP and standard was 19.80 and 12.81 μg/mL, respectively. The IC_50_ values of free polyphenol and bound polyphenol extracts from red date peel for scavenging DPPH radicals were 72.58 ± 3.82, 54.45 ± 5.98 μg/mL, respectively (Dou et al., 2023). In contrast, RBSDF-BP had significantly greater scavenging capacity for DPPH free radicals. The phenolic compounds quantified in this study were abundant, including many flavonols, which were effective in scavenging free radicals. The antioxidant activity of flavonols was largely dependent on their highly conjugated chemical structures and specific hydroxylation patterns. Phenolic acids containing aromatic hydroxylated rings that can provide quenching single-linear state oxygen and hydrogen atoms played a crucial part in their antioxidant activity (Camboim Rockett et al., 2020).

The FRAP method was generally used to assess the ability to reduce Fe^3+^ to Fe^2+^, with greater FRAP values indicating better antioxidant ability. As shown in Table 6, the corresponding IC_50_ values were 86.39, 58.20 μg/mL. Based on FRAP, ABTS^+^• and DPPH• experimental data, RBSDF-BP showed good antioxidant activity in terms of reducing power and free radical scavenging. It may have been mainly attributed to the existence of numerous phenolic compounds, such as epicatechin, catechin and gallic acid, which could be good electron donors for free radical scavenging or reducing effects owing to the large amount of hydroxyls.

### 3.5 Inhibitory effect of RBSDF-BP with *α*-glu

#### 3.5.1 Inhibitory activities on *α*-glu

**Fig. 5:**
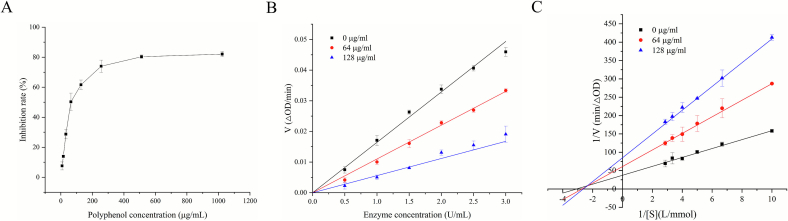
The inhibiting activity of RBSDF-BP with *α*-glu. (A) Impact of RBSDF-BP content on inhibiting rate; (B) The kinetic curve of *α*-glu inhibiting by RBSDF-BP; (C) The Lineweaver-Burk plot of *α*-glu inhibiting by RBSDF-BP.

This study utilized yeast *α*-glu to evaluate in vitro inhibitory activity, which serves as a universal model for efficient preliminary screening. In order to further characterize the biological activity of RBSDF-BP in a specific aspect, a comprehensive analysis of its inhibitory effects on *α*-glu was performed. The inhibition of *α*-glu by polyphenols was shown in Fig. 5A. The extracts inhibited *α*-glu in a concentration-dependent manner over the concentration range of 2 μg/mL to 1024 μg/mL, at a highest inhibition level of 82.07 % and an IC_50_ value of 84.25 μg/mL. When the concentration of RBSDF-BP was greater than 512 μg/mL, the inhibition no longer showed a substantial increase. This inhibitory potency is notably superior to that of the classic drug acarbose (IC₅₀ = 196.31 μg/mL) (Xing et al., 2024), highlighting the potential of RBSDF-BP as a natural *α*-glu inhibitor. Unfermented beans, fermented beans, unfermented liquor, and fermented liquor cocoa polyphenols extract effectively inhibited *α*-glu minimum IC_50_ = 90.0 μg/mL (Ryan et al., 2016). In contrast, RBSDF-BP also displayed more significant inhibitory activity of *α*-glu.

#### 3.5.2 Inhibition type and constant of RBSDF-BP

Based on the binding mechanism of the inhibitor to the enzyme, inhibitory actions are categorized into reversible and irreversible modes. Fig. 5B showed the kinetic curves of *α*-glu inhibition by RBSDF-BP. The straight lines fitted to the points at the three concentrations passed by the origin and the slopes were inversely related to the enzyme concentration, revealing that it was a classic reversible inhibition. In the pattern, small molecules interact with the enzyme through noncovalent bonds and can physically restore *α*-glu activity.

The main categories of enzyme inhibition were competitive, non-competitive, uncompetitive, and mixed inhibition. The inhibitory effect of RBSDF-BP on *α*-glu was further characterized by determining the Michaelis constant (Km) utilizing the Lineweaver-Burk plot, which employs a double reciprocal equation. As depicted in Fig. 5C, the linearly fitted extended lines were found to coincide at a point in the second quadrant. With increasing inhibitor concentration (0–128 μg/mL), the Michaelis constant (K_m_) increased progressively as the maximal velocity (V_max_) decreased. This suggested that RBSDF-BP was mixed-type inhibitors of *α*-glu, which would mean that RBSDF-BP bind to both free *α*-glu and the *α*-glu-PNPG complex. Acarbose is a typical competitive inhibitor, whereas the bound polyphenol extracted from *Phyllanthus emblica* Linn (PEBP). acts as a mixed-type inhibitor of *α*-glu (Xing et al., 2024). Compared to competitive inhibitors, this mixed-type inhibition mechanism may enable PEBP to effectively suppress *α*-glu activity across a broader range of substrate concentrations, which could be one reason for the stronger inhibitory effects of PFBP and RBSDF-BP.

### 3.6 Fluorescence quenching analysis

**Fig. 6:**
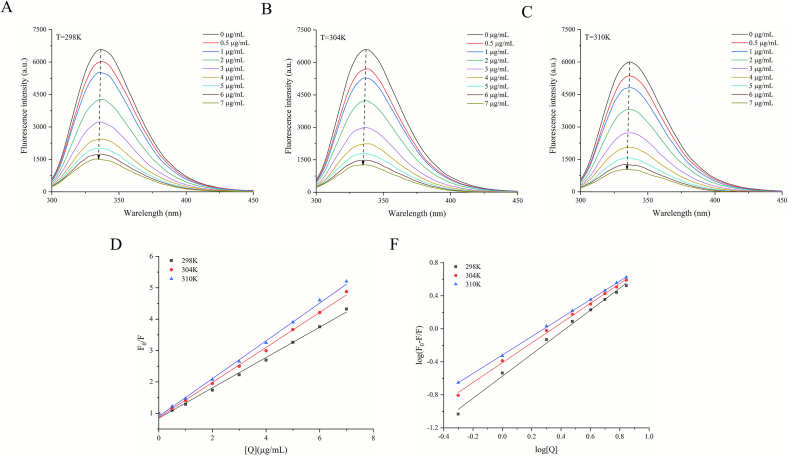
Impact of RBSDF-BP on diverse concentrations on the intrinsic fluorescence of *α*-glucosidase at 298 K (A)、304 K (B) and 310 K (C); (D) The Stern-Volmer plot of fluorescence quenching of *α*-glucosidase through RBSDF-BP; (E) Plot of log [*F*_*0*_—*F*)/*F*] with log [*Q*] for fluorescence quenching of *α*-glucosidase through RBSDF-BP.

Fluorescence quenching is typically employed to describe the reduction of fluorescence quantum yield and can effectively illuminate the conformation of enzymes and their interactions with inhibitors. The *α*-glu is endowed with fluorescent properties by three chromophores, tyrosine (Tyr), tryptophan (Trp), and phenylalanine (Phe), and all of them form an emission spectrum at an excitation wavelength of 280 nm, which is the sum of the fluorescence emitted by *α*-glu (Yin et al., 2023). Therefore, in order to study the interaction between RBSDF-BP and *α*-glu, we measured the fluorescence intensity of *α*-glu before and after adding RBSDF-BP at an excitation wavelength of 280 nm. Fig. 6A-C revealed the effect of different concentrations of RBSDF-BP on the fluorescence of *α*-glu at 298, 304, 310 K. The strongest fluorescence emission peak emerged near 337 nm, and the indole group in the tryptophan residue was the major fluorophore. At all three temperatures, RBSDF-BP significantly reduced the intrinsic fluorescence intensity of *α*-glu in a concentration-dependent way. The apparent fluorescence quenching results show that the endogenous fluorescence of *α*-glu was counteracted by different levels of quenching agents and that there was an obvious interaction between the *α*-glu chromophore and RBSDF-BP. Meanwhile, the fluorescence emission peak of *α*-glu shifted from the initial 343.4 nm to 330.8 nm with a blue shift in the presence of RBSDF-BP. This indicated that the polarity of the microenvironment of the tryptophan residue of *α*-glu decreased and the hydrophobicity increased. In summary, RBSDF-BP effectively bound to *α*-glu and caused a conformational variation in *α*-glu.

Commonly, the fluorescence quenching mechanisms can be divided into static quenching, dynamic quenching and the combination of these two quenching forms. As shown in Fig. 6D and Table 7, the R^2^ values of the quench curves were > 0.99 at three different temperatures, indicating that the linear relationship was well fitted and that there existed a class of fluorophores during the binding process, which was all prone to collide with RBSDF-BP or form complexes. Since RBSDF-BP was a mixture, the relative molecular mass of the most abundant protocatechuic acid was used as a standard to calculate Kq. It had been shown that the quenching rate constant Kq was greater than the rate constant of maximum diffusive collisional quenching of biomolecules by all types of quenching agents [2 × 10^10^ L·mol^−1^·s^−1^], which suggested that the quenching mechanism was mainly a static quench. The findings were similar to the reported fluorescence quenching effect was similar to that of procyanidin dimer on *α*-glu which also follows a static quenching mechanism (Dai et al., 2019). The contribution of hydrophobic interactions can be reflected by Table 7, which showed that the *K*_*SV*_ value increased gradually with the increase of temperature from 298 K to 318 K. In short, if hydrophobic interactions dominate, they were enhanced by hydrophobic forces under heating to the extent of increasing the probability of complex formation.

**Table 7:** Quenching constants and binding parameters of RBSDF-BP with *α*-glu at different temperature.

*T* (K)	*K*_*SV*_ (L/mg)	*K*_*q*_(10^12^L/(mol·s)	*R*_*a*_^*2*^	*K*_*a*_(10^−1^ L·mg^−1^)	*n*	*R*_*b*_^*2*^
298	0.48 ± 0.01^c^	7.43	0.9956	2.66 ± 0.05^c^	1.33 ± 0.02^a^	0.9957
304	0.56 ± 0.03^b^	8.58	0.9973	3.88 ± 0.05^b^	1.19 ± 0.01^a, b^	0.9980
310	0.61 ± 0.02^a^	9.35	0.9982	4.81 ± 0.06^a^	1.11 ± 0.04^b^	0.9998

a, b, c represent significantly different (*P* < 0.05).

Double logarithmic curves were made to obtain the apparent binding constant and the quantity of binding sites (Fig. 6F). We found that the binding constant of RBSDF-BP to the enzyme increased with the increase of temperature, which was favorable for the generation of the complex and indicated that the process was a heat-absorbing reaction. In addition, in Table 7 it can be found that at three temperatures (298 K, 304 K, 310 K) the binding site n value is about 1, indicating a class of binding sites between RBSDF-BP and *α*-glu, which is consistent with the prediction of the binding site of kaempferol to *α*-glu (Gong et al., 2023).

### 3.7 Synchronous fluorescence

**Fig. 7:**
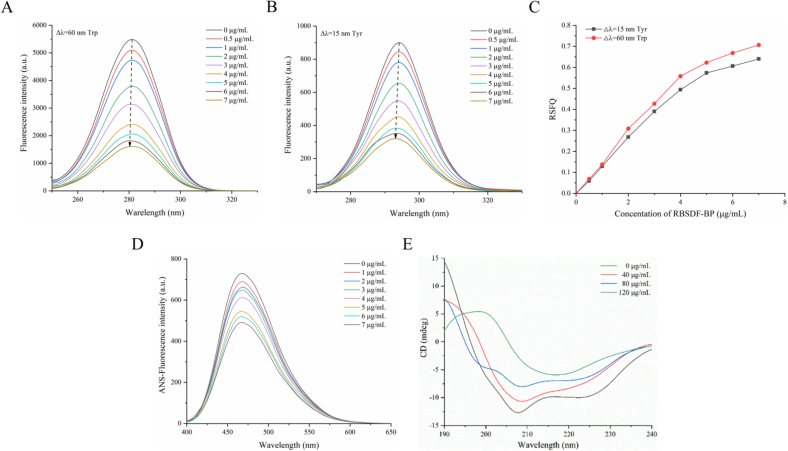
Synchronous fluorescence spectra of *α*-glucosidase with RBSDF-BP at Δλ = 60 nm (A) and Δλ = 15 nm (B); (C) The RSFQ plots for Tyr and Trp. (D) ANS-fluorescence spectra of *α*-glucosidase with RBSDF-BP; (E) CD spectra of *α*-glucosidase with RBSDF-BP.

The corresponding amino acids are tyrosine and tryptophan when the maximum emission wavelength migration Δλ of the excitation and emission wavelengths is fixed at 15 nm and 60 nm, respectively. Synchronized fluorescence was introduced at this time to respond to changes in the polar microenvironment around Tyr and Trp residues (Sun et al., 2024). Fig. 7A-B demonstrated the synchronized fluorescence spectra of *α*-glu upon the addition of different concentrations of RBSDF-BP. The results displayed that the fluorescence intensity of the tryptophan residue was significantly higher than that of the Tyr residue, which further proved that the intrinsic fluorescence of *α*-glu mainly originated from the tryptophan residue. The Tyr and Trp residues fluorescence intensity weakened with increasing concentrations of RBSDF-BP, and the maximum emission wavelengths showed a slight blue shift from 294.4 nm to 292.6 nm and from 280.8 nm to 279.2 nm, respectively, indicating that the microenvironment near tyrosine and tryptophan residues is less polar and more hydrophobic.

In addition, the ratio of synchronized fluorescence quench RSFQ was calculated simultaneously by Eq. 5, and the Trp and Tyr residues fluorescence quench intensities were decreased by 70.64 % and 63.99 %, when the concentration of RBSDF-BP attained 7 μg/mL. The RSFQ values at Δλ = 60 nm were all higher than the corresponding values at Δλ = 15 nm (Fig. 7C). This indicated that the tryptophan residues seem to contribute more to the fluorescence quenching effect of the binding process than the Tyr residues. It could be hypothesized that RBSDF-BP might have been closer to the tryptophan when bound to the catalytic center of the enzyme.

### 3.8 Surface hydrophobicity analysis

The surface hydrophobicity of the RBSDF-BP-*α*-glu complex was determined by fluorescence spectroscopy using a non-covalent fluorescent probe, ANS, which is able to bind to the hydrophobic regions of proteins through non-covalently bound hydrophobic interactions. As could be seen from the results (Fig. 7D), the surface hydrophobicity of *α*-glu gradually decreased with ascending RBSDF-BP concentration. It indicated that the binding of RBSDF-BP reduced the hydrophobic solvent accessible surface area of *α*-glu. This might have been due to the fact that polyphenols can act as ligands to cover the hydrophobic regions on the enzyme surface through non-covalent interactions, thus reducing the binding sites of ANS. Studies on the addition of polyphenols to reduce the proteins surface hydrophobicity have shown the same conclusions, such as myofibrillar protein (Cheng et al., 2020), lactoferrin and whey protein.

### 3.9 Circular dichroism (CD) spectra analysis

The conformational changes of *α*-glu induced by RBSDF-BP binding in the far UV region (190–250 nm) were further assessed with circular dichroism spectroscopy (Gong et al., 2023). The CD spectra in the 190–240 nm region and secondary structures were shown in Fig. 7E and Table 8, respectively.

**Table 8:** Secondary structure content after reaction with *α*-glucosidase using several concentrations of RBSDF-BP.

RBSDF-BP (μg/mL)	*α*-Helix (%)	*β*-Sheet (%)	*β*-Turns (%)	Rondom coil
0	31.0 ± 0.8^a^	11.9 ± 0.6^d^	19.9 ± 0.8^c^	37.3 ± 0.4^a^
40	23.0 ± 0.6^b^	16.1 ± 0.4^c^	24.8 ± 0.3^b^	36.2 ± 0.5^a^
80	17.5 ± 0.3^c^	21.0 ± 0.3^b^	27.6 ± 0.5^a^	34.0 ± 0.8^b^
120	13.7 ± 0.5^d^	25.2 ± 0.2^a^	28.9 ± 0.6^a^	32.2 ± 0.4^c^

a, b, c, d represent significantly different (*P* < 0.05).

The CD plot of *α*-glu showed two significant negative peaks at 208 nm and 220 nm, which are indicative of the presence of the *α*-helical structure (Feroz et al., 2012). The strong peak recorded at 208 nm was caused by the π → π * jump, and the reduction of the negative peak observed after the addition of RBSDF-BP indicates a change in the *α*-helical component of the protein (Zeng et al., 2016). It also appeared to affect *β*-sheet, which normally showed one negative peak at 215 nm. The results displayed that the *α*-helix content decreased from 31.0 % to 13.7 % with increasing concentration of RBSDF-BP. On the contrary, the percentage of *β*-sheet content increased from 11.9 % to 25.2 %, and the percentage of *β*-turn increased from 19.9 % to 28.9 %. Random coils decreased from 37.3 % to 32.2 %. The proportion of *α*-helix in *α*-glu was further reduced after incubation with RBSDF-BP. This may lead to a loosening of the enzyme structure, thus affecting the formation of the active center. Hydrogen bonds maintain the stability of the proteins secondary structure, and changes in the secondary structure reveal a corresponding change in the site of hydrogen bond formation (Zhao et al., 2020).

The increase in *β*-sheet content with the addition of RBSDF-BP suggests that RBSDF-BP may disrupt the hydrogen bond network, compromising the stability of the conformation. Meanwhile, the increase of the *β*-turn content and the decrease of the random curl content indicated that RBSDF-BP changed the spatiality conformation of the enzyme by inducing rearrangement of the *α*-glu secondary structure, thus affecting the binding of the enzyme active site to the substrate. Wang et al. found that the content of the *α*-helix component of *α*-amylase decreased sharply upon binding to Chinese bayberry leaves proanthocyanidins (BLPs) and the *β*-sheet content increased significantly, indicating that the BLPs induced *α* -amylase structure was more loose and unstable compared to the natural state (Wang et al., 2020).

Thus, it can be concluded that the binding of RBSDF-BP to the enzyme tends to result in partial unfolding and loosening of *α*-glu, which further hinders the binding of the substrate to *α*-glu and ultimately affects the enzyme activity (Ren et al., 2024). The above results may confirm the findings of fluorescence spectroscopy and contribute to the understanding of the mechanism by which RBSDF-BP inhibits *α*-glu activity.

### 3.10 Molecular docking analysis

**Fig. 8:**
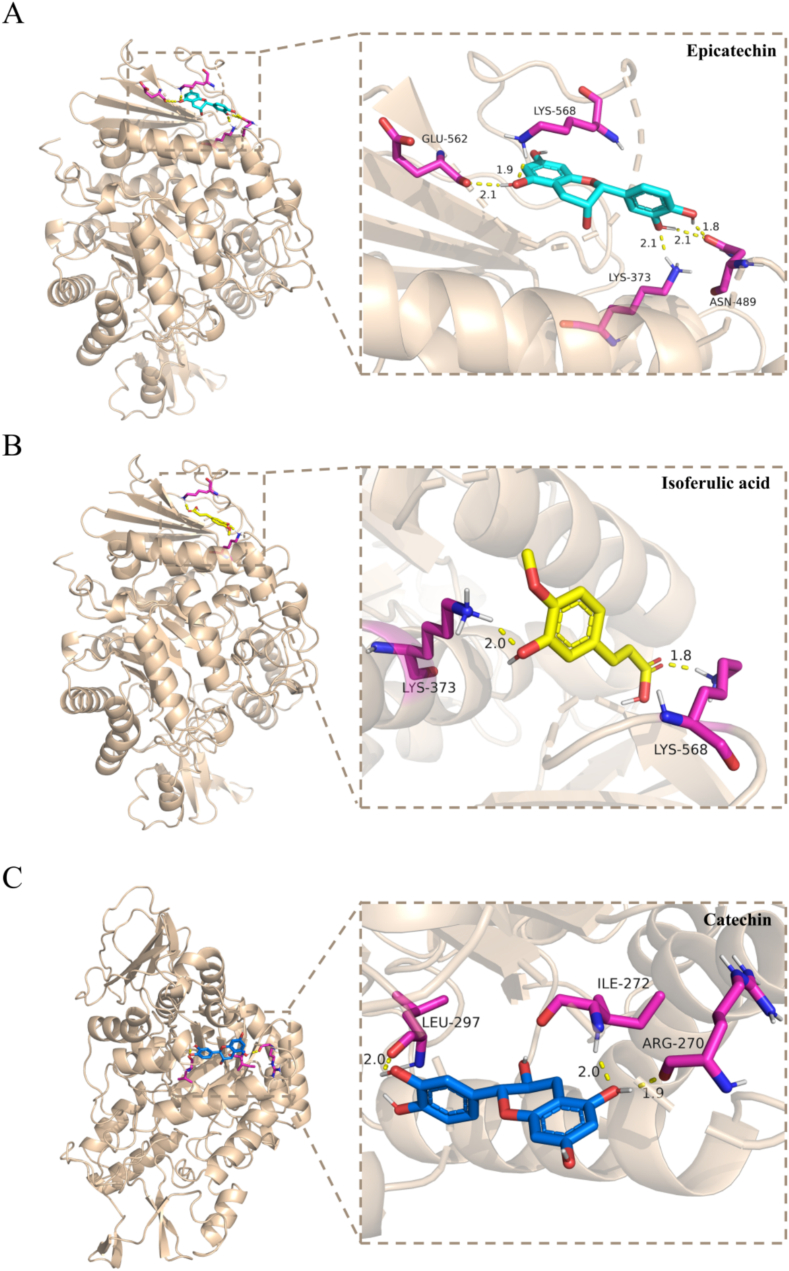
The optimal docking result and interaction mode of *α*-glucosidase with epicatechin (A), isoferulic acid (B) and catechin (C), respectively.

In order to further clarify the inhibition mechanisms, the top ten polyphenols with the highest RBSDF-BP content were molecularly docked with *α*-glu, and the three polyphenols with the lowest binding energies were finally selected and plotted and analysed by pymol. The characterization of binding energies, binding sites and binding forces are shown in Table 9. The optimal docking results of epicatechin, isoferulic acid and catechin with *α*-glu are shown in Fig. 8. Due to the presence of hybrid inhibition between RBSDF-BP and the enzyme with intricate interactions, blind docking was used in this study to identify the docking regions.

**Table 9:** The non-covalent interactions between polyphenol ligands and *α*-glucosidase.

Ligands	Affinity (kcal/mol)	Interaction types and relevant residues	
		Hydrogen bonds	Hydrophobic interactions
Epicatechin	−6.81	Lys373 (2.1 Å), Asn489 (1.8 Å, 2.1 Å), Glu562 (2.1 Å), Lys568 (1.9 Å)	Lys568 (1.9 Å)
Isoferulic acid	−5.89	Lys373 (2.0 Å), Lys568 (1.8 Å)	Phe494 (1.8 Å)
Catechin	−6.30	Arg270 (1.9 Å), Ile 272 (2.0 Å), Leu297 (2.0 Å)	Glu296 (1.9 Å)

Based on the molecular docking results, the optimal binding energies of epicatechin, isoferulic acid, and catechin docked with *α*-glu were − 6.81, −5.89, and − 6.30 kcal/mol, respectively. In addition, all binding energies were negative, indicating that the binding of polyphenols to the enzymes was an exothermic process. Epicatechin was surrounded by the 13 amino acids of *α*-glu and formed five hydrogen bonds with ASN489, GLU562, GLY564, and LYS568 through the hydroxyl groups on the side chain of the ab ring at an average distance of 2 Å. Of these, LYS568 was involved in the formation of hydrophobic interactions (Fig. 8A). As shown in Fig. 8B, amino acid residues LYS373 and LYS568 of *α*-glu interacted with isoferulic acid to form two hydrogen bonds with bond distances of 2.0 and 1.8 Å, respectively. Among them, PHE494 was involved in the formation of the π-π T-shaped interaction, which was also a hydrophobic interaction. As shown in Fig. 8C, catechin formed three hydrogen bonds with amino acid residues ARG270, ILE272 and LEU297 of *α*-glu via hydroxyl groups on the side chain of the ab ring with distance range values of 1.9–2.0 Å, respectively, and formed an amide-π stacked interaction with GLU296, which was also classified as a hydrophobic interaction. Previous reports have also identified additional π-alkyl interactions of catechin with *α*-glu (PDB: 5NN5) (Tiji et al., 2021).

In general, lower docking scores indicated a more stable ligand-receptor complex, suggesting a higher potential for inhibition. The lowest binding energy of epicatechin could have been attributed to more hydrogen bonding and hydrophobic forces at the binding site of epicatechin with *α*-glu (Zhang et al., 2024). Hydrogen bonding and van der Waals force were also discovered to be the major forces involved in the interaction between tea polyphenols and *α*-glu. In addition, the benzene ring of isoferulic acid was perpendicularly conjugated to the aromatic ring of Phe. However, the extent of this overlap was weak compared to the typical π-π stacking effect, as it was not a parallel overlap of the two rings (Li et al., 2025). The binding energy of isoferulic acid was the highest of the three, probably due to the low number of hydrogen bonds and the weak hydrophobic interaction.

Catechin has been shown to be competitively inhibited with *α*-glu, acting in the active pocket (Zhang et al., 2024). By taking up the binding part of the enzyme active center, the ligand led to competitive inhibition of the substrate while altering the microenvironment of *α*-glu, resulting in a decrease in *α*-glu activity. LYS373 and LYS568 were both involved in the hydrogen bonding of isoferulic acid and epicatechin to the enzyme, indicating that both phenolic compounds may interact with other binding site of *α*-glu and exhibit a non-competitive or mixed type of inhibition (Xu et al., 2024). These results confirmed the judgment that the type of inhibition in the kinetics was mixed inhibition and circular dichroism conformational change. Although the 3A4A was widely used for the preliminary screening of natural inhibitors, and its predictive results were validated in this study through enzyme inhibition assays, the sequence similarity indicated potential differences in the active site microenvironment compared to mammalian α-glu. Therefore, studies employing proteins with higher homology were considered necessary for more precise simulations in the future.

In summary, multiple components of RBSDF-BP including epicatechin, isoferulic acid and catechin were potential ligands for *α*-glu. These findings could explain how RBSDF-BP interacted mainly with key amino acid residues through non-covalent forces, docked to the active site of the *α*-glu, changed the conformation of the *α*-glu, and exerted their inhibitory effects on the *α*-glu. These results suggested that RBSDF-BP may have potential as an inhibitor of *α*-glu and for the prevention of hyperglycaemia and its related complications.

## 4 Conclusion

The present study focused on the content, composition, antioxidant properties and mechanism of *α*-glu inhibitory activity of bound polyphenols from rice bean skin dietary fiber. The results showed that the polyphenol content of alkaline hydrolysed extract (52.54 mg GAE/g DM) was higher than that of acidic and enzymatically hydrolysed extracts. A sum of 44 polyphenolic compounds were identified, of which 17 main components were quantitatively analysed. RBSDF-BP showed significant scavenging impacts on DPPH• and ABTS^+^• radicals and reducing ability to ferric ions. The results showed that RBSDF-BP reversibly inhibited *α*-glu in a concentration-dependent mixed inhibition type. The *α*-glu bound to RBSDF-BP showed a decrease in surface hydrophobicity and a change in conformation mainly from *α*-helix and random helix to β-fold and β-sheet. The molecular docking results indicated that hydrogen bonding and hydrophobic force were the major non-covalent bonding factors for the binding interaction of RBSDF-BP with *α*-glu. These results suggested that RBSDF-BP may have potential as an inhibitor of *α*-glu and for the prevention of hyperglycaemia and its related complications. RBSDF-BP can be added as a functional ingredient to glucose-lowering foods to avoid the risk of side effects associated with traditional drugs. The application of RBSDF-BP in complex food systems (e.g., yogurt, beverages) necessitates stability assessment in model systems. Furthermore, the bioavailability and in vivo functional activity of RBSDF-BP require further validation through systematic evaluation in insulin-resistant mouse models.

## CRediT authorship contribution statement

**Jing Liang:** Writing – original draft, Visualization, Methodology, Investigation, Formal analysis, Conceptualization. **Jiayan Xie:** Supervision, Methodology, Investigation, Conceptualization. **Yue Gu:** Validation, Resources, Methodology, Investigation. **Jianhua Xie:** Supervision, Project administration. **Yi Chen:** Supervision, Project administration. **Bing Zheng:** Methodology, Investigation, Data curation. **Yue Qiu:** Validation, Methodology, Investigation. **Xiaobo Hu:** Supervision, Project administration. **Qiang Yu:** Writing – review & editing, Supervision, Resources, Project administration, Funding acquisition.

## Funding

This work was supported by the 10.13039/501100001809National Natural Science Foundation of China (31972066).

## Declaration of competing interest

The authors declare that they have no known competing financial interests or personal relationships that could have appeared to influence the work reported in this paper.

## Data Availability

Data will be made available on request.

## Appendix A Supplementary data

Supplementary material Image 1
