# Supplementary material for: Optimization and identification of bound polyphenols from rice bean (Vigna umbellata) skin dietary fiber, antioxidant activities and α-glucosidase inhibition mechanism
Source: Food Chem X. 2025 Nov 8;32:103278. doi: 10.1016/j.fochx.2025.103278 (PMC12657305; doi:10.1016/j.fochx.2025.103278)
Supplement: Supplementary file 1 — Supplementary material [file mmc1.docx]

**Supplementary materials**

**Table S1**

Design of single factor experiment for releasing of total polyphenol by alkaline hydrolysis.

| single factor | NaOH concentration(M) | Liquid-solid ratio (mL/g) | Time (h) |
| --- | --- | --- | --- |
| NaOH concentration | 4, 6, 8, 10, 12. | 15 | 3 |
| Liquid-solid ratio | 10 | 5, 10, 15, 20, 25. | 3 |
| Time | 10 | 15 | 1, 2, 3, 4, 5. |

**Table S2**

Design of single factor experiment for releasing of total polyphenol by acid hydrolysis.

| single factor | H_2_SO_4_ concentration (%) | Liquid-solid ratio (mL/g) | Time (h) |
| --- | --- | --- | --- |
| H_2_SO_4_ concentration | 2, 7, 12, 17, 22. | 15 | 3 |
| Liquid-solid ratio | 12 | 5, 10, 15, 20, 25. | 3 |
| Time | 12 | 15 | 1, 2, 3, 4, 5. |

**Table S3**

Design of single factor experiment for releasing of total polyphenol by enzymatic hydrolysis.

| single factor | Enzymatic concentration (mg/mL) | Liquid-solid ratio (mL/g) | Time (h) |
| --- | --- | --- | --- |
| Enzymatic concentration | 0.6, 1.2, 1.8, 2.4, 3.0. | 15 | 3 |
| Liquid-solid ratio | 1.8 | 5, 10, 15, 20, 25. | 3 |
| Time | 1.8 | 15 | 1, 2, 3, 4, 5. |

**Table S4**

Design and results of Box-Behnken experiment in acid hydrolysis.

| Number | H_2_SO_4_  concetration (X1) | Liquid-solid  ratio (X2) | time (X3) | TPC (Y) |
| --- | --- | --- | --- | --- |
| 1 | 7(-1) | 10(-1) | 3(0) | 4.354 |
| 2 | 17(1) | 10(-1) | 3(0) | 5.029 |
| 3 | 7(-1) | 20(1) | 3(0) | 4.843 |
| 4 | 17(1) | 20(1) | 3(0) | 4.650 |
| 5 | 7(-1) | 15(0) | 2(-1) | 4.307 |
| 6 | 17(1) | 15(0) | 2(-1) | 4.679 |
| 7 | 7(-1) | 15(0) | 4(1) | 3.587 |
| 8 | 17(1) | 15(0) | 4(1) | 3.810 |
| 9 | 12(0) | 10(-1) | 2(-1) | 4.478 |
| 10 | 12(0) | 20(1) | 2(-1) | 4.828 |
| 11 | 12(0) | 10(-1) | 4(1) | 3.708 |
| 12 | 12(0) | 20(1) | 4(1) | 3.777 |
| 13 | 12(0) | 15(0) | 3(0) | 6.500 |
| 14 | 12(0) | 15(0) | 3(0) | 6.285 |
| 15 | 12(0) | 15(0) | 3(0) | 6.200 |
| 16 | 12(0) | 15(0) | 3(0) | 6.404 |
| 17 | 12(0) | 15(0) | 3(0) | 6.214 |

**Table S5**

Design and results of Box-Behnken experiments in enzymatic hydrolysis.

| Number | Enzymatic concetration (X1) | Liquid-solid ratio (X2) | time (X3) | TPC (Y) |
| --- | --- | --- | --- | --- |
| 1 | 1.2(-1) | 10(-1) | 3(0) | 6.159 |
| 2 | 2.4(1) | 10(-1) | 3(0) | 6.411 |
| 3 | 1.2(-1) | 20(1) | 3(0) | 6.508 |
| 4 | 2.4(1) | 20(1) | 3(0) | 6.233 |
| 5 | 1.2(-1) | 15(0) | 2(-1) | 6.263 |
| 6 | 2.4(1) | 15(0) | 2(-1) | 6.550 |
| 7 | 1.2(-1) | 15(0) | 4(1) | 6.677 |
| 8 | 2.4(1) | 15(0) | 4(1) | 6.512 |
| 9 | 1.8(0) | 10(-1) | 2(-1) | 6.221 |
| 10 | 1.8(0) | 20(1) | 2(-1) | 6.056 |
| 11 | 1.8(0) | 10(-1) | 4(1) | 6.086 |
| 12 | 1.8(0) | 20(1) | 4(1) | 6.414 |
| 13 | 1.8(0) | 15(0) | 3(0) | 7.721 |
| 14 | 1.8(0) | 15(0) | 3(0) | 7.768 |
| 15 | 1.8(0) | 15(0) | 3(0) | 7.704 |
| 16 | 1.8(0) | 15(0) | 3(0) | 7.708 |
| 17 | 1.8(0) | 15(0) | 3(0) | 7.681 |

**Table S6**

ANOVA for the quadratic model in acid hydrolysis.

| source | Sum of Squares | df | Mean Square | F | p |  |
| --- | --- | --- | --- | --- | --- | --- |
| Model | 16.62 | 9 | 1.85 | 149.32 | < 0.0001 | significant |
| X1 | 0.145 | 1 | 0.145 | 11.72 | 0.0111 |  |
| X2 | 0.035 | 1 | 0.035 | 2.83 | 0.1365 |  |
| X3 | 1.45 | 1 | 1.45 | 117.52 | < 0.0001 |  |
| X1X2 | 0.1884 | 1 | 0.1884 | 15.23 | 0.0059 |  |
| X1X3 | 0.0056 | 1 | 0.0056 | 0.4488 | 0.5244 |  |
| X2X3 | 0.0197 | 1 | 0.0197 | 1.6 | 0.2469 |  |
| X1^2^ | 3.06 | 1 | 3.06 | 247.01 | < 0.0001 |  |
| X2^2^ | 2.37 | 1 | 2.37 | 191.4 | < 0.0001 |  |
| X3^2^ | 7.94 | 1 | 7.94 | 641.83 | < 0.0001 |  |
| Residual | 0.0866 | 7 | 0.0124 |  |  |  |
| Lack of Fit | 0.0203 | 3 | 0.0068 | 0.4073 | 0.7567 | not significant |
| Pure Error | 0.0663 | 4 | 0.0166 |  |  |  |
| Cor Total | 16.71 | 16 |  |  |  |  |

**Table S7**

ANOVA for the quadratic response surface model in enzymatic hydrolysis.

| source | Sum of Squares | df | Mean Square | F | p |  |
| --- | --- | --- | --- | --- | --- | --- |
| Model | 7.11 | 9 | 0.7898 | 568.58 | < 0.0001 | significant |
| X1 | 0.0012 | 1 | 0.0012 | 0.882 | 0.3789 |  |
| X2 | 0.0139 | 1 | 0.0139 | 10.04 | 0.0157 |  |
| X3 | 0.0449 | 1 | 0.0449 | 32.29 | 0.0007 |  |
| X1X2 | 0.0694 | 1 | 0.0694 | 49.98 | 0.0002 |  |
| X1X3 | 0.0511 | 1 | 0.0511 | 36.77 | 0.0005 |  |
| X2X3 | 0.0608 | 1 | 0.0608 | 43.74 | 0.0003 |  |
| X1² | 1.23 | 1 | 1.23 | 887.83 | < 0.0001 |  |
| X2² | 3.02 | 1 | 3.02 | 2176.92 | < 0.0001 |  |
| X3² | 1.92 | 1 | 1.92 | 1379.86 | < 0.0001 |  |
| Residual | 0.0097 | 7 | 0.0014 |  |  |  |
| Lack of Fit | 0.0056 | 3 | 0.0019 | 1.78 | 0.2896 | not significant |
| Pure Error | 0.0042 | 4 | 0.001 |  |  |  |
| Cor Total | 7.12 | 16 |  |  |  |  |

**Table S8**

The predicted and actual value of response in acid hydrolysis.

|  | H_2_SO_4_ concentration (%) | Liquid-solid ratio (mL/g) | Extraction time (h) | Total polyphenol content (mg GAE/g IDF) |
| --- | --- | --- | --- | --- |
| Optimum conditions (predicted) | 12.39 | 15.20 | 2.84 | 6.36 |
| Modified conditions (actual) | 12.00 | 15.00 | 3.00 | 6.40 |

**Table S9**

The predicted and actual value of response in enzymatic hydrolysis.

|  | Enzymatic concentration (mg/mL) | Liquid-solid ratio (mL/g) | Extraction time (min) | Total polyphenol content (mg GAE/g IDF) |
| --- | --- | --- | --- | --- |
| Optimum conditions (predicted) | 1.39 | 18.56 | 3.18 | 7.14 |
| Modified conditions (actual) | 1.20 | 20.00 | 3.00 | 7.06 |


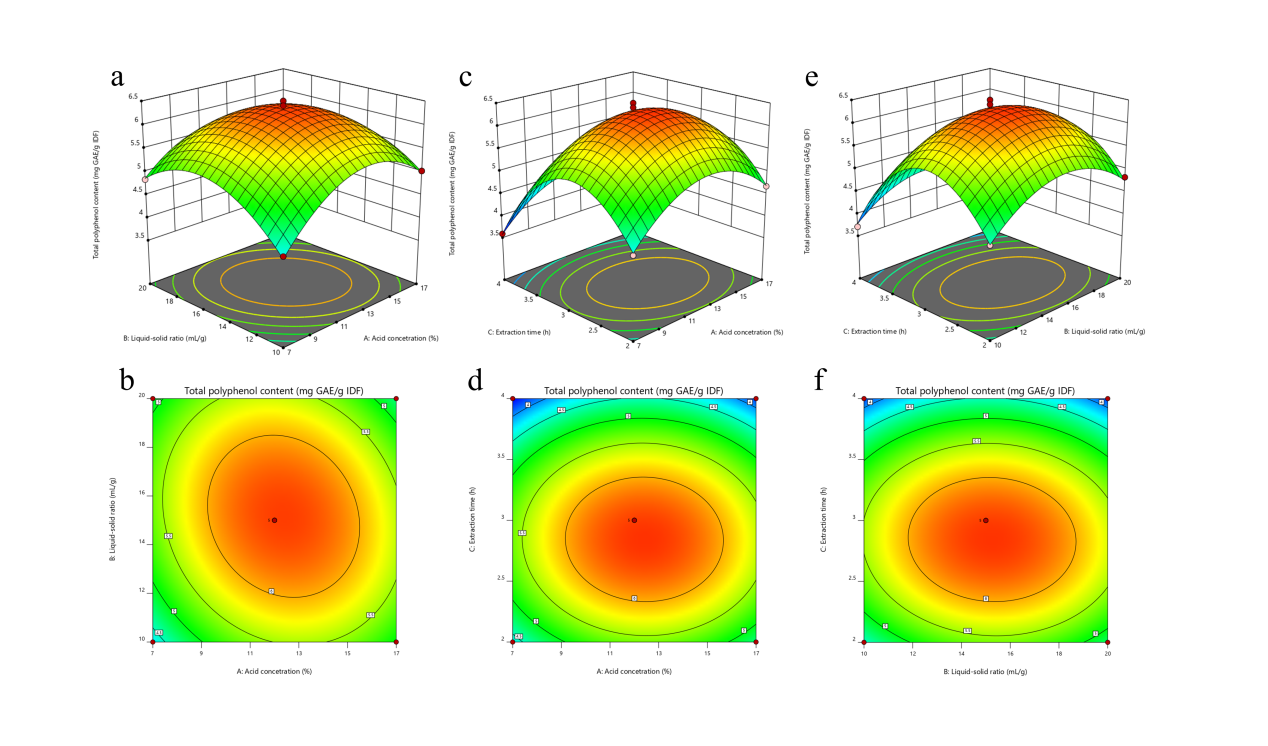


**Fig.S1.** Response surface plots. H_2_SO_4_ concentration and liquid-solid ratio (a and b). H_2_SO_4_ concentration and extraction time (c and d). Liquid-solid ratio and extraction time (e and f).


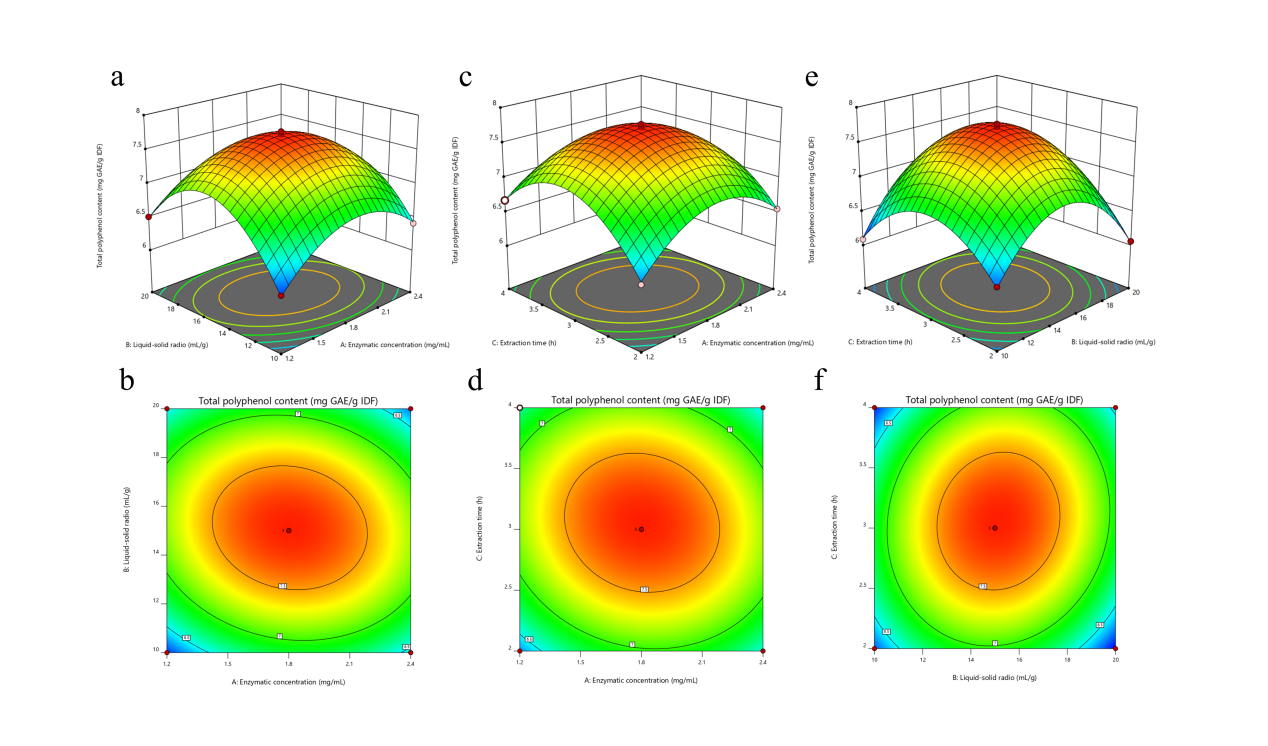


**Fig.S2.** Response surface plot and contour plot of variables and their mutual interactions. Enzymatic concentration and liquid-solid ratio (a and b). Enzymatic concentration and extraction time (c and d). Liquid-solid ratio and extraction time (e and f).


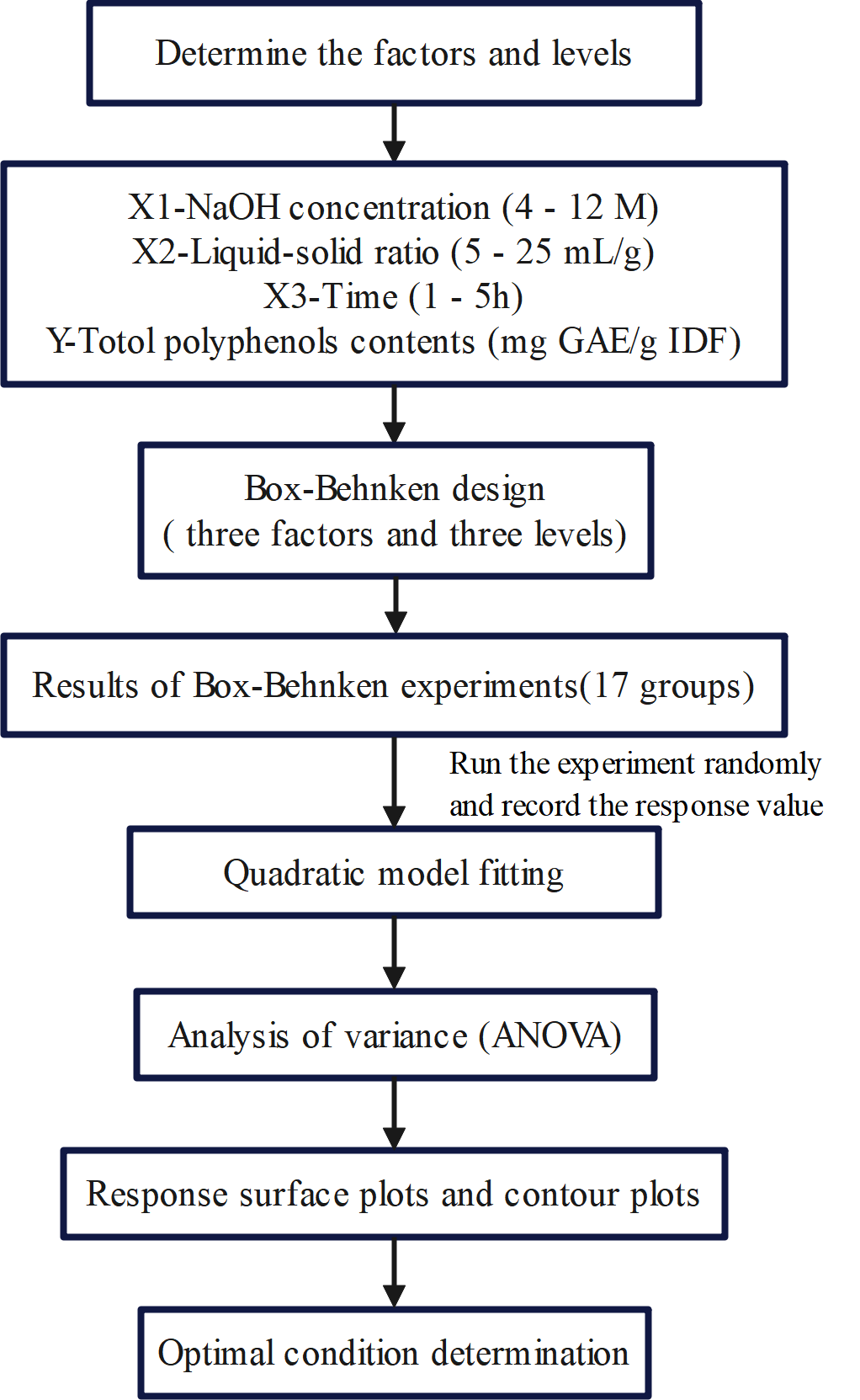


**Fig.S3.** Box-Behnken design flowchart.
